# The Quality of Dying in Frail Institutionalized Older Patients After Nonoperative and Operative Management of a Proximal Femoral Fracture: An In-Depth Analysis

**DOI:** 10.1177/10499091231180556

**Published:** 2023-07-05

**Authors:** Sverre A. I. Loggers, Romke Van Balen, Hanna C. Willems, Taco Gosens, Suzanne Polinder, Kornelis J. Ponsen, Cornelis L. P. Van de Ree, Jeroen Steens, Michael H. J. Verhofstad, Rutger G. Zuurmond, Pieter Joosse, Esther M. M. Van Lieshout

**Affiliations:** 1 Department of Surgery, 1140 Northwest Clinics Alkmaar, Alkmaar, the Netherlands; 2 Trauma Research Unit, Department of Surgery, 6993 Erasmus MC, University Medical Center Rotterdam, Rotterdam, the Netherlands; 3 Department of Public Health and Primary Care, 4501 Leiden University Medical Center, Leiden, the Netherlands; 4 Geriatrics Section, Department of Internal Medicine, 26066 Amsterdam UMC Location AMC, Amsterdam, the Netherlands; 5 Department of Orthopaedic Surgery, 7898 Elisabeth-TweeSteden Ziekenhuis, Tilburg, the Netherlands; 6 Department of Public Health, 6993 Erasmus MC, University Medical Center Rotterdam, Rotterdam, the Netherlands; 7 Department of Surgery, Rode Kruis Ziekenhuis, Beverwijk, the Netherlands; 8 Department Trauma TopCare, 7898 Elisabeth-TweeSteden Ziekenhuis, Tilburg, the Netherlands; 9 Department of Orthopaedic Surgery, 3956 Dijklander Ziekenhuis, Hoorn, the Netherlands; 10 Department of Orthopaedic Surgery, 8772 Isala, Zwolle, the Netherlands

**Keywords:** quality of dying, proximal femoral fracture, palliative care, quality of dying and death, hip fracture, nonoperative

## Abstract

Proximal femoral fractures in frail patients have a poor prognosis. Despite the high mortality, little is known about the quality of dying (QoD) while this is an integral part of palliative care and could influence decision making on nonoperative- (NOM) or operative management (OM). To identify the QoD in frail patients with a proximal femoral fracture. Data from the prospective FRAIL-HIP study, that studied the outcomes of NOM and OM in institutionalized older patients ≥70 years with a limited life expectancy who sustained a proximal femoral fracture, was analyzed. This study included patients who died within the 6-month study period and whose proxies evaluated the QoD. The QoD was evaluated with the Quality of Dying and Death (QODD) questionnaire resulting in an overall score and 4 subcategory scores (Symptom control, Preparation, Connectedness, and Transcendence). In total 52 (64% of NOM) and 21 (53% of OM) of the proxies responded to the QODD. The overall QODD score was 6.8 (P_25_-P_75_ 5.7-7.7) (intermediate), with 34 (47%) of the proxies rating the QODD ‘good to almost perfect’. Significant differences in the QODD scores between groups were not noted (NOM; 7.0 (P_25_-P_75_ 5.7-7.8) vs OM; 6.6 (P_25_-P_75_ 6.1-7.2), P = .73). Symptom control was the lowest rated subcategory in both groups. The QoD in frail older nursing home patients with a proximal femoral fracture is good and humane. QODD scores after NOM are at least as good as OM. Improving symptom control would further increase the QoD.

## Introduction

A proximal femoral fracture in frail older patients is a devastating injury and often the start of a cascade breakdown at the end of life. In addition to the already limited life expectancy of 58% in the first year after admission for nursing home residents, the injury results in a profound decrease in functional ability and quality of life (Qol).^1^ Previous studies have shown that operative management (OM) in frail older patients has poor outcomes.^2,3^ Distressing adverse events and delirium occur very frequently and many nursing home patients become immobile after the fracture, with a high mortality rate due to a variety of reasons.^2,4,5^ These poor outcomes raise the question whether surgery for frail nursing home patients with poor pre-fracture life expectancy facing a proximal femoral fracture is the most favorable management strategy. In patients with dementia, 60% of the proxies of nursing home residents feel that only treatments that promote comfort would best align with their goals of care.^6^ This places a more palliative care approach in the center of the clinical management of frail older nursing home residents with a limited life expectancy.^7^ Consequently, this often means relinquishing from life-sustaining treatments.^6^ For proximal femoral fractures this means considering both operative and nonoperative management (NOM). NOM in properly selected patients has shown to be noninferior to OM with regard to (health-related) quality of life ((HR)QoL) and is associated with high treatment satisfaction.^5^ Therefore, NOM with focus on comfort and QoL with adequate analgesia could also be a viable alternative.

The important question is whether patients with a proximal femoral fracture can have an acceptable palliative process that supports these patients in their final phase of life with a good quality of dying (QoD). The current literature fails to address this important aspect,^8,9^ despite the fact that 13% out of 1.3 million annual patients who sustain a proximal femoral fracture worldwide die within 30 days.^10,11^ A high-quality dying process is reasonably free of discomfort, in accordance with patients’ wishes, and within acceptable professional and ethical standards. A good QoD is an integral part of palliative care.^12^

Because improving care at the end of life requires an accurate assessment of the experienced dying process, this aspect of the care of frail older patients who sustain a proximal femoral fracture remains to be elucidated. This accounts especially for nonoperative managed patients, since relieve of discomfort is often used as an argument for surgical intervention, despite exposing patients to additional risks such as surgical complications, delirium, and pneumonia.

The current study performed an in-depth analysis of the QoD after NOM and OM in a population of frail older institutionalized patients who sustained a proximal femoral fracture.

## Methods

This in-depth analysis of the QoD used data collected during the FRAIL-HIP study. This multicenter prospective cohort study evaluated the effect of NOM vs OM in a selected group of institutionalized frail older patients with a limited life expectancy who sustained a proximal femoral fracture. The study was registered at the Netherlands Trial Register (NTR7245). The study protocol of this study has been published previously.^13^ The Medical Research Ethics Committee of VUmc approved the study (2018.208)

Frail institutionalized older patients (ie, 70 years or older, living in a nursing home pre trauma) that sustained a proximal femoral fracture (femoral neck AO-type 31-B.1 to .3 or AO-type 31-A.1.2 to .3) who either; were malnutrition (Body Mass Index (BMI) of <18.5 km/m^2^ or cachexia), had existing mobility issues (Functional Ambulation Category (FAC) of 2 or less), or had severe comorbidities (American Society of Anesthesiologists (ASA) class 4 or 5) were included. Patients with subtrochanteric, bilateral, or peri-prosthetic fractures, proximal femoral fractures with a delay of diagnosis of more than 7 days, known metastatic disease, or insufficient comprehension of the Dutch language or who participated in another surgical intervention or drug trial that could have influenced results were excluded. Patients received care as usual throughout the study period and were followed for 6 months.

Between September 1, 2018 and April 25, 2020, 172 patients were included in the FRAIL-HIP study of whom 88 opted for NOM and 84 for OM after a process of shared decision making (SDM). Treatment decision was reached after discussing the goals of care and pros and cons of both NOM and OM with patients, their relatives or legal representatives (proxies), and all relevant health care providers involved.

For the present study only those patients who died during their study participation and whose proxies responded to the QoD questionnaire were included. The primary outcome of this analysis was the quality of dying as measured by the Quality of Dying and Death (QODD) Questionnaire.

The QODD a is 17-item interview-based questionnaire on 17 end-of-life priorities.^14^ It is the most often used, the best validated instrument on the quality of dying.^15^ The QODD questionnaire was conducted after the death of the patients and the timing depended on the proxies preference for conducted the questionnaire.

Each of the 17-items includes a filter question if or to what extent items occurred during the final period of the decedent’s life from a patient-proxy perspective. This is followed by a rating to score the quality of the item on a scale from 0 to 10, with 0 representing a ‘terrible’ experience to 10 representing an ‘almost perfect’ experience from a proxy-proxy perspective. Besides an overall score on the QODD, 4 subscales can be distinguished. These 4 categories are symptom control (ie, pain control, control over what was going on, and ability to breathe comfortably), preparation (ie, having means to hasten death if desired, visiting with spiritual advisor, having funeral arrangements in order, avoiding life support, and having health care costs covered), connectedness (ie, sharing physical expressions of affection and spending time with family and friends) and transcendence (ie, feeling unafraid of dying, feeling at peace with dying, and feeling untroubled about strain on loved ones). Scores are calculated by dividing the total sum of scores by the number of completed items from the rating questions. The scores are categorized into the categories ‘terrible to poor’ (0-2.9), ‘intermediate’ (3-6.9), and ‘good to almost perfect’ (7-10). This applies to both the subscales and overall score, but not to the separate items.^16^

### Analysis

Data were analyzed using the Statistical Package for the Social Sciences (SPSS) version 25.0 (SPSS, Chicago, Ill., USA), and are reported following the Strengthening the Reporting of Observational studies in Epidemiology (STROBE) guidelines. The analyses were performed on a per-protocol basis. Normality of continuous data was tested with the Shapiro-Wilk test. Continuous data were reported as median and quartiles and categorical data as number with percentage. Univariate comparison between the groups was done using a Mann-Whitney *U*-test, Chi-squared test or Fisher’s Exact test (as applicable). A two-sided P-value <.05 was taken as threshold of statistical significance.

## Results

### Patient- and Fracture Characteristics

**Figure 1.**
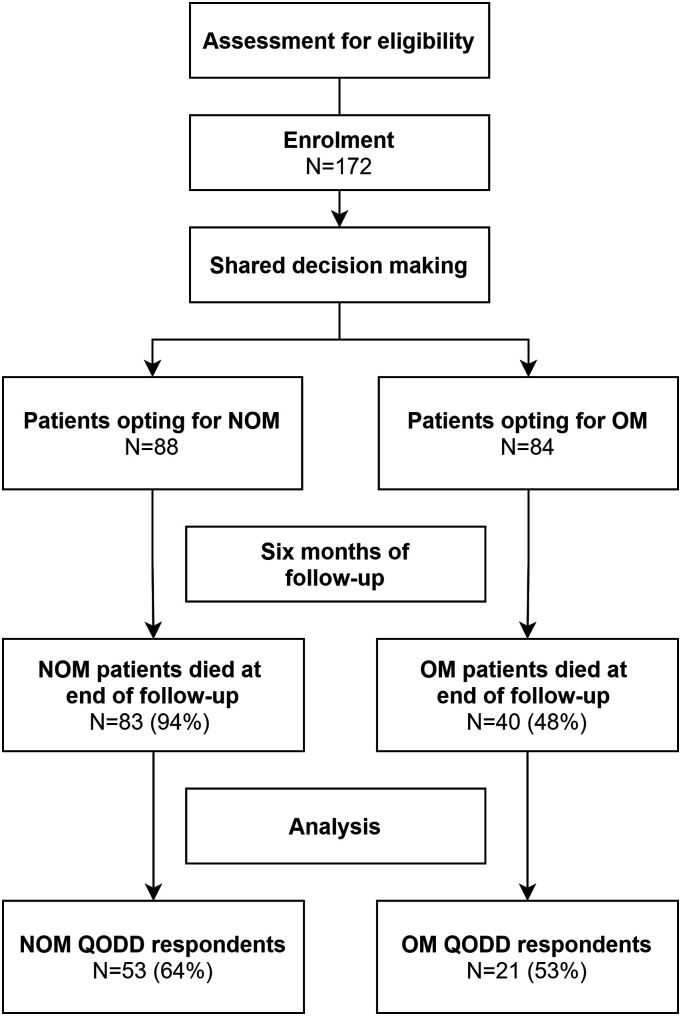
Flow of participants and number of patients in analysis. NOM, nonoperative management; OM, operative management; QODD, Quality of dying and death.

During the study period 83 (94%) patients of the NOM group and 40 (48%) of the OM group died. In total, 53 (64%) proxies in the NOM group and 21 (53%) in the OM group responded to the QODD and were included in the final analysis (Figure 1).

The median age of the included patients was 89 years (P_25_-P_75_ 86-93) and 52 (70%) were female. A total of 68 (92%) patients suffered from dementia. There were no statistically significant differences in patient- and fracture characteristics between responders and non-responders to the QODD in the NOM or OM group. Out of all proxy QODD responders 62 (84%) were son/daughter, 6 (8%) partner, 2 (3%) brother/sister, 2 (3%) a close friend, 1 (1% sibling) and 1 (1%) a non-related legal representative. There was no statistical difference in the responding proxies’ relation to the patient between the NOM and OM group (P = .241). After opting for NOM 24 (45%) patients returned to their nursing home residency without hospital admission. In-hospital palliative care teams were consulted in 5 (8%) patients in the NOM and 1 (5%) in the OM group.

### Characteristics of Death

Three patients in the NOM group (6%) and 2 (10%) in the OM group died in hospital. All other patients in both groups died in their own nursing home residency, besides 1 patient (5%) in the OM group who was transferred to a hospice upon request of the patient and family. There were no statistically significant differences in the place of death between the 2 groups (P = .226). In the NOM group 24 (46%) and 7 (33%) in the OM group received palliative sedation (at the discretion of the treating physician) in the final stage of the dying process (P = .316). The median time to death was shorter in the NOM group (7.0 days (P_25_-P_75_ 4.5-12)) than in the OM group (25.0 days (P_25_-P_75_ 12.5-59.5; P < .001).

### QODD Rating

**Figure 2.**
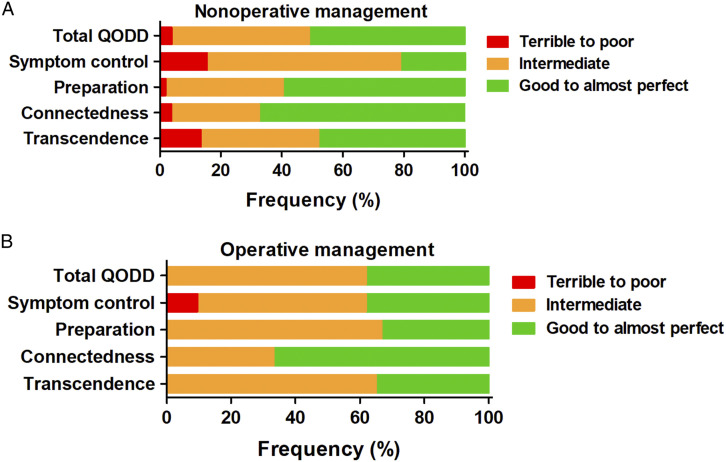
Distribution of frequencies with the categorical ratings of the total QODD score and 4 subscales. A: scores of the nonoperative management group. B: Scores from the operative management group. Frequencies are reported as percentages (%) of total respondents. QODD, Quality of Dying and Death.

The overall rated QODD in the NOM group was rated as ‘good to almost perfect’ by 26 proxies (51%), ‘intermediate’ by 23 (45%) and ‘terrible to poor’ by 2 proxies (4%) (Figure 2). For the OM group, this was ‘good to almost perfect’ in 8 (38%) and ‘intermediate’ in 13 (62%) patients (Figure 2). The ‘symptom control’ domain was the lowest rated out of the 4 subcategories in both groups. No statistical differences were found in the median rating between the NOM and OM group for the total score or any of the subdomains (Table 1). Also, no statistical differences for the categorical ratings between the NOM and OM group were found for the QODD with regards to the overall score (.733), symptom control (P = .120), preparation (P = .150), connectedness (P = .864) or transcendence (P = .550) (Table 1).

**Table 1.** Rating of the Separate Aspects, Subdomains, and Overall Quality of Dying and Death Score for the Nonoperative and Operative Group.

Item	Nonoperative	Operative	P-value
Symptom control	**5.6 (3.1-6.7)**	**6.4 (4.3-7.5)**	.120
Had control of pain	5.5 (2.3-7.0)	7.0 (5.0-8.0)	.104
General control	5.0 (2.0-7.0)	5.5 (2.5-7.0)	.526
Breath comfortably	6.0 (3.0-8.0)	7.0 (5.0-7.0)	.339
Preparation	**7.0 (6.0-8.0)**	**6.4 (5.4-7.6)**	.150
Means to hasten death	6.0 (3.0-8.0)	6.0 (5.0-7.0)	.938
Visit spiritual advisor	8.0 (5.5-9.0)	6.0 (5.0-8.0)	.051
Arranged funeral	7.0 (5.0-9.0)	7.0 (5.0-8.0)	.395
Avoiding life support	8.0 (5.0-9.0)	7.0 (6.0-8.0)	.928
Health care costs covered	8.0 (7.0-9.3)	8.0 (7.0-8.0)	.486
Connectedness	**7.5 (6.0-9.0)**	**7.5 (6.0-8.5)**	.864
Physical expression of affection	8.0 (7.0-9.0)	8.0 (7.0-8.0)	.316
Spend time with family	7.0 (5.0-8.0)	8.0 (6.0-9.0)	.995
Transcendence	**6.7 (4.5-8.0)**	**6.2 (4.4-7.6)**	.550
At peace with dying	7.0 (5.0-8.0)	6.0 (5.0-8.0)	.584
Unafraid of dying	7.0 (5.0-8.0)	6.5 (5.0-8.0)	.545
Strain on loved ones	7.0 (5.0-8.0)	6.0 (4.0-7.8)	.544
Other	N.A.	N.A	N.A.
Control of bladder or bowels	5.0 (2.0-7.0)	5.0 (4.0-6.0)	.449
Laugh and smile	6.0 (4.0-7.0)	6.0 (4.0-7.0)	.949
Keep dignity and self-respect	5.0 (3.0-8.0)	6.0 (4.3-7.8)	.302
Said goodbye to loved ones	5.0 (3.0-8.0)	5.5 (3.3-8.0)	.785
Financial problems	8.0 (7.5-10.0)	8.0 (7.0-8.0)	.187
Overall QODD score	**7.0 (5.7-7.8)**	**6.6 (6.1-7.2)**	.733

Numbers are presented as median (P_25_-P_75_).

QODD, Quality of Dying and Death; N.A., not applicable.

### Frequency of Occurrence of QODD Items

**Figure 3.**
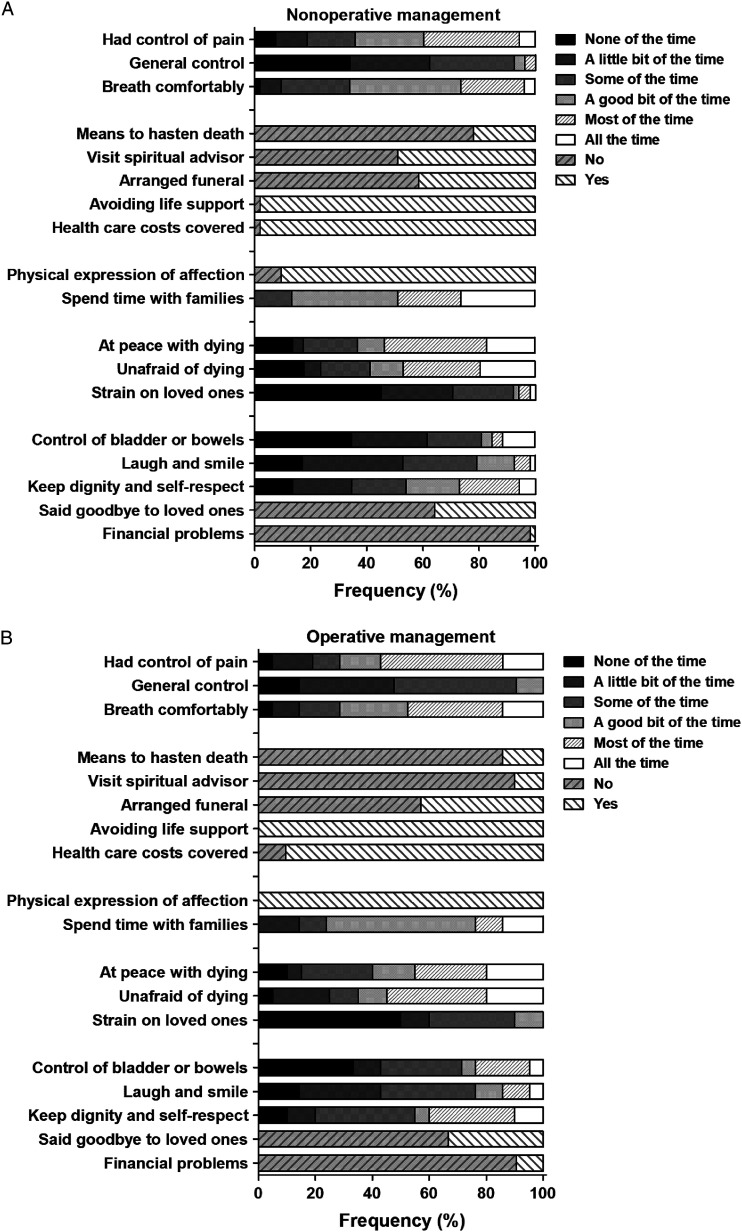
Percentages of if- or to what extent items occurred during the final period of the decedent’s life. A: scores of the nonoperative management group. B: Scores from the operative management group. Numbers are shown as percentage (%) of total respondents.

The percentage if or to what extent items of the QODD occur during the final period the decedent’s life in both groups is displayed in Figure 3. There were no statistically significant differences between the NOM and OM group with regards to if, or how often certain aspects occurred, besides that the NOM group patients spent more time with their family compared to the OM group (P = .028).

A large proportion of patients in both groups (NOM; 34 (64%), OM; 14 (67%)) did not get to say goodbye to loved ones prior to their death. The question concerning if patients had their funeral arrangements in order was positively answered in 42% of the NOM patients and 43% of the OM patients.

Breathing was adjudged comfortable in the majority of cases. Pain control was similar in both groups. However, 36% of the patients in the NOM group and 29% in the OM group pain was controlled in ‘none-to ‘some of the time’.

### Relationship Between Characteristics and QODD Outcomes

There was a statistically higher total QODD score for patients in the NOM group who died within 30 days (47 (89%)) vs those who died after 30 days (6 (11%)). The median score of the patients who died within 30 days was 7.1 (P_25_-P_75_ 5.8-8) and who died later was 5.5 (P_25_-P_75_ 2.4-6.8) (P = .047). These differences were not seen for other timeframes (7 days, 14 days, or 3 months) or in the OM group for all timeframes. For those patients who died within 30 days, no statistical difference in total QODD score between the NOM and OM group was found (NOM; 7.1 (P_25_-P_75_ 5.8-8), OM; 6.8 (P_25_-P_75_ 6.2-7.3, P = .765). For the overall QODD score there were no statistically significant differences between males and females (P = .483), fracture type (P = .051), or whether or not patients received palliative sedation (P = .174).

## Discussion

This study showed that overall the QoD in frail patients with a proximal femoral fracture was good and humane, based on the large proportion of high QODD scores in both groups. Almost half of the proxies rated the QoD as ‘good to almost perfect’ and only a very small proportion (3%) rated the QoD as low. The QoD in the NOM group was at least as good as the QoD in the OM group. For frail nursing home residents with a limited life expectancy who sustain a proximal femoral fracture, this means that the argument to routinely opt for OM instead of NOM in order to improve the QoD is most likely invalid. The QoD could be further improved, by optimizing symptom control in both groups. Especially pain control would require optimization, since in one-third of the patients’ pain was not to sometimes adequately controlled according to proxies. In case death is believed to be imminent, supportive care with adequate analgesia to support the dying process is of the utmost importance as the data suggest that delaying death leads to a poorer QoD. This could be explained by a prolonged period of pain and immobility and therefore discomfort. Initial symptom control in-hospital could be improved by performing ultrasound guided nerve blocks with prompt results whereby the initial 24-48 hours period could be bridged in which an adequate oral/intramuscular analgesia management strategy could be initiated.

To our knowledge, this is the first study to address the QoD using the QODD questionnaire in proximal femoral fracture patients. No QODD scores were found for patients with any other trauma related injuries. A limited number of previous studies have described results in non-trauma related patient populations. Gerritsen et al. have described a QODD in ICU patient in the Netherlands (median 8 (P_25_-P_75_ 7-9), and Darer et al. in a general sample including patients with end-stage renal disease, congestive heart failure, and chronic obstructive pulmonary disease (median 7.4 (P_25_-P_75_ 5.7-8.6).^17,18^ Lower mean scores of 5.5 (SD 1.5) in patients with cancer in Israel have also been reported.^19^

As mentioned, a good QoD is an integral part of palliative care.^12^ The role of palliative care is well established in treatment of other disease like cancer, chronic cardiac failure, chronic obstructive pulmonary disease, and diabetes.^20,21^ Early integration of palliative care of patients with these diseases is associated with improved continuity and coordination of care, greater quality of life, and improved symptom control.^20–22^ Because of the deleterious impact of proximal femoral fractures on mortality and functional outcomes, previous studies have also suggested that a more palliative care approach to frail older patients with a proximal femoral fracture is indicated.^23^ However, despite the positive influence of palliative care on QoL and symptom control in patients with other diseases, evidence of the impact of early integrative palliative care for proximal femoral fracture patients is scarce. Current guidelines only recognize that surgery can be considered as a palliative measure, but do not provide support or evidence for early palliative care referrals or selection criteria for nonoperative management.^8,9^ As Harries et al. have pointed out in their study on attitudes and approaches to palliative care in patients with a proximal femoral fracture, a palliative care approach should not be seen as a failure of active management but as an adjunct to holistic care.^24^ Early integration of palliative care should therefore be seen as an anticipation that could only contribute to the quality of life and treatment satisfaction.

However, it must be realized that unlike chronic conditions with a long disease course, a proximal femoral fracture is an acute event which could lead to a period of clinical decline with severely decreased QoL. Currently, curation and rehabilitation are the main goals in frail patients with a proximal femoral facture. In older patients that are not properly screened for frailty the need for geriatric palliative care might not be recognizable until the postoperative or rehabilitation period, especially in case no advance care planning (ACP) is done prior to the injury. In case the frailty of patients is underestimated and consequently these goals are proven not be achievable this would cause a delay in optimal symptom control and supportive care and could lead to a worse QoD. Therefore, carefully considering all treatment options is vital in frail patients with a limited life expectancy.

A key part of palliative care in this group of patients is therefore the early recognition of patients that require a more palliative care centered approach instead of intense rehabilitation. Based on the QoD results, there are no differences in the QoD in patients that die within 30 days after the injury between NOM and OM. Although the sample size was limited, it could therefore be suggested that for considering NOM with regards to the QoD, a patient’s life expectancy should not exceed 30 days.

More importantly, this study also reiterates the importance of goals of care discussions and the role of advance care planning in long term care facilities.^25^ ACP in nursing home residents decreases unwanted “aggressive,” harmful or non-beneficial treatments like hospitalization, surgery, and life support.^26,27^ This study provides unique data to further support ACP for decision making for operative or nonoperative management and possible ‘do no hospitalize’ directives in case of a proximal femoral fracture in the most frail nursing home residents with a limited residual quantity- and quality of life.

In this study in-hospital palliative care teams were rarely consulted. Previous studies on palliative care team consultation and QoD have displayed favorable effects on the QODD, mainly due to the improved anticipation and relatives being able to say goodbye.^28^ The low rate of palliative care team consultation is explained by the major role of elderly care physicians in long term care facilities in the Netherlands. In the Netherlands elderly care physicians are trained and dedicated to care in nursing homes including palliative- and rehabilitative care. Palliative care team consultation in nursing homes is therefore generally not required in the Netherlands and only used in the hospital- or community setting.

Symptom control, and pain control in particular was the lowest rated subcategory of the QoD. Other studies have also shown that pain is prevalent in dying geriatric patients. In addition these studies also found that there is considerable variability in use and dosage of supportive analgesia in palliative geriatric patients that can be improved.^29–31^ Initial symptom control in-hospital could be improved by performing ultrasound guided nerve blocks with quick results (within 30 min) whereby the initial 24-48 hours period could be bridged in which an adequate oral/intramuscular analgesia management strategy could be initiated that sufficiently supports symptoms management and therefore improves the QoL and QoD of these patients in the final days of life.

### Limitations

This study has some limitations. Beside the smaller sample size, only limited characteristics of the proxies were collected. Therefore, the respondent’s characteristics could not be correlated to the QoD, although more than 80% was son or daughter. The QODD response rate of 64% for the NOM and 53% for the OM group may possible have resulted in some selection bias. However, patient characteristics did not differ between responders and non-responders therefore reducing minimizing this effect. In addition, these response rates are higher than some previous studies evaluating the QODD.^19,32^ Furthermore, since this was a secondary analysis of existing data, the study was not primarily powered to detect statistical differences between the NOM and OM group for the QODD and therefore reducing the strength of the findings. At last, correction for multiple variables in order the check for confounder was not deemed feasible due to the limited sample size.

However, despite the limitations, this study is the first to address the QoD in this specific group of frail patients who died at a median of 10 days after sustaining a proximal femoral fracture. Current literature has scarce data to guide proxy decision makers regarding short- and long-term outcomes of NOM and OM in frail older patients with a proximal femoral fracture. Because of the poor prognosis of frail institutionalized older patients who sustain a proximal femoral factor, next to the prefracture QoL, the QoD could be a decisive factor for opting for NOM or OM. The results of this study show that the QoD for NOM is at least as good as that for OM, reassuring that NOM results in an acceptable QoD. In this way it provides supportive data for expectation management, ACP, and ultimately decision making for NOM vs OM when health care providers engage in goals-of-care and end-of-life discussions with frail older patients with a proximal femoral fracture.

## Conclusion

The rating of the quality of dying in frail older nursing home patients with a pre-fracture limited life expectancy who sustain a proximal femoral fracture is good and humane. The results suggest that no major differences were observed in the quality of dying in nonoperatively treated patients compared to operatively treated patients. Improving symptom control, and especially pain, could be a mean to further increase the quality of dying in both groups.
